# Purtscher-like retinopathy and paracentral acute middle maculopathy following breast filler injection

**DOI:** 10.1186/s12886-023-03186-8

**Published:** 2023-11-07

**Authors:** Xu Kent Pee, Adeline Low, Mas Edi Putriku Intan Ab Kahar, Shelina Oli Mohamed, Ying-Jiun Chong

**Affiliations:** 1 Department of Ophthalmology, Sarawak General Hospital, 93000 Kuching, Malaysia; 2 Department of Ophthalmology, Shah Alam Hospital, 40000 Shah Alam, Malaysia; 3 Department of Ophthalmology, Faculty of Medicine, Universiti Teknologi MARA, Sungai Buloh Campus, 47000 Sungai Buloh, Selangor, Malaysia

**Keywords:** Paracentral acute middle maculopathy, Purtscher-like retinopathy, Breast filler injection, Hyaluronic acid

## Abstract

**Background:**

To report a rare case of pulmonary and ocular complications with visual loss due to bilateral Purtscher-like retinopathy and paracentral acute middle maculopathy (PAMM) following a hyaluronic acid (HA) filler injection to the breast. Systemic and visual recovery was attained following corticosteroid therapy.

**Case presentation:**

A 27-year-old lady presented with painless blurring of vision in both eyes for 2 weeks following hyaluronic acid breast filler injections by a non-medical practitioner. She was initially admitted to the medical ward for diffuse alveolar haemorrhage and altered sensorium. The presenting visual acuity was counting fingers in both eyes. Bilateral dilated fundus examination showed hyperaemic discs, concentric rim of retinal whitening around macula with patches of polygonal-shaped retinal whitening, generalised cotton-wool spots, tortuous veins, and flame-shaped haemorrhages. Spectral-domain optical coherence tomography (SD-OCT) macula revealed hyper-reflective bands at the inner nuclear layer (INL). Fluorescein angiography demonstrated hot discs, delayed arm-to-retina time, arterial filling, and arterio-venous transit time with staining of the vessels at the posterior pole. She was managed with a tapering dose of systemic corticosteroids. The visual acuity improved to 6/12 over 8 weeks with significant anatomical and functional improvement. Dilated fundus examination showed resolution of initial funduscopy findings. The hyper-reflective bands on the OCT had resolved with subsequent thinning of the INL and disorganisation of retinal inner layers.

**Conclusion:**

Filler injections are in increasing demand and are frequently being performed by non-medical practitioners. Visual loss from non-facial HA fillers is rare. Inadvertent entry of HA into a blood vessel may potentially cause systemic and sight-threatening ocular complications. Good anatomical knowledge and proper injection technique are vital in preventing this unfortunate sequela. There are limited reports on successful visual recovery following various treatment approaches and we hope this case provides valuable insights.

## Background

Cosmetic filler injection is a common aesthetic procedure to increase dermal tissue volume. It has gained popularity as a minimally invasive procedure to increase tissue volume for enhancement of appearance. The composition of these fillers can be collagen, hyaluronic acid (HA), polylactic acid, calcium hydroxyapatite and polymethylmethacrylate (PMMA) [1]. The American Society for Aesthetic Plastic Surgery reported that HA is the preferred cosmetic filler due to its ease of administration, safety profile and favourable outcomes [2].

Filler injections rarely cause sight-related adverse events but if they do occur, they are frequently irreversible resulting in catastrophic complications. The mechanism for this is inadvertent retrograde embolisation with the entrance of filler materials into the ophthalmic circulation and central retinal artery through the external carotid-internal carotid anastomoses [3]. In a systematic review of post-HA filler-associated visual loss, 44 cases all of which involved facial HA fillers were identified [4].

In contrast, non-facial fillers have a lower incidence of blinding ocular complications. As HA injected into the subdermal region is usually a blind procedure, the direction and angle of the needle during injection are hence crucial as there is a possibility of inadvertent entry of the needle into a blood vessel, causing an intraluminal inoculation of HA [5].

There are several reported cases of filler-associated visual loss, and almost all cases involved injection into facial tissues. To our best knowledge, visual loss following filler injection to breast tissue has yet to be reported. We report a rare case of systemic complications and visual loss secondary to bilateral Purtscher-like retinopathy and PAMM following a HA filler injection to the breast. Systemic and visual recovery was achieved with corticosteroid therapy.

## Case report

A 27-year-old healthy female was admitted to the medical ward for sudden onset of shortness of breath and reduced responsiveness immediately after undergoing an HA filler injection to her right breast by a non-medical practitioner. The estimated volume of injection was around 500 millilitres. She was diagnosed with acute respiratory distress syndrome. Initial laboratory investigations upon admission showed leukocytosis at 13.7 × 10^3^/µL with otherwise normal haemoglobin and platelet counts. The erythrocyte sedimentation rate was 18 mm/hour. Other blood investigations including renal profile, liver function, coagulation profile, serology for syphilis, hepatitis B & C and HIV, and autoimmune panel were all unremarkable.

The contrast-enhanced computed tomography (CECT) thorax showed multiple imperceptible tiny nodular-like lesions in both breasts, diffuse bilateral ground-glass opacification, and consolidative changes in both lungs predominantly at the upper lobes and at the periphery. Bronchoscopy and bronchoalveolar lavage confirmed features of diffuse alveolar haemorrhage. An echocardiography was attempted but there was limited visualisation due to poor penetration. She was also noted to have altered sensorium and CECT brain showed a left corona radiata infarct with no radiographic evidence of intracranial haemorrhage. There were intermittent generalised rhythmic delta waves suggestive of diffuse cerebral encephalopathy on the electroencephalography suggestive of toxic encephalopathy. The patient did not consent to a lumbar puncture. She was managed with intravenous ceftriaxone and hydrocortisone by the physicians after which her condition stabilised, and she was discharged well after two weeks with a tapering dose of oral corticosteroids.

**Fig. 1:**
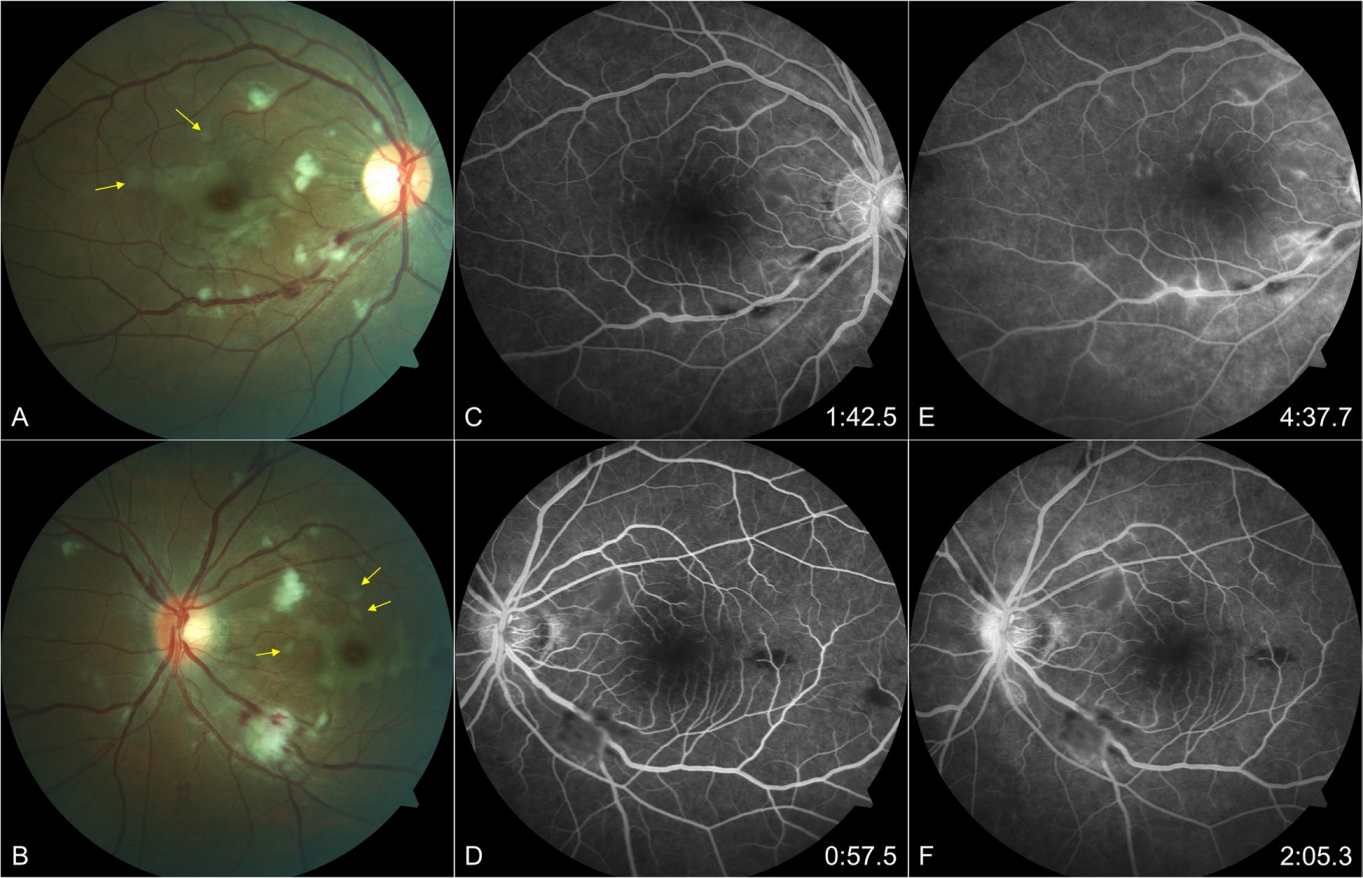
Fundus photograph of right and left eye (**A**, **B**) showing tortuous veins, multiple cotton wool spots and retinal haemorrhages mainly in the posterior pole in keeping with Purtscher’s like retinopathy. Note the concentric rim of perifoveal retinal whitening and a few patches of polygonal-shaped retinal whitening (yellow arrows) **C–F**: FFA showing delayed venous filling of right superotemporal vein (at 1:42 min), staining of both optic discs, masking from cotton wool spots and haemorrhages, perifoveal vasculitis, and vasculitis of vessels in the posterior pole, enlarged foveal avascular zone and capillary non-perfusion perifoveally in both eyes

Although she had also experienced sudden bilateral painless visual loss within 24 h after the procedure, she was only referred to the Ophthalmologist two weeks later upon discharge from the medical ward. She denied eye redness, discomfort, seeing floaters or flashes. Upon presentation to the Ophthalmologist, the visual acuity was counting fingers in both eyes. There was no relative afferent pupillary defect. She could not perceive any numbers on Ishihara’s colour vision plates. Anterior segment examination was unremarkable. Dilated fundus examination of the right eye showed a pink optic disc and multiple small to large cotton wool spots distributed mainly at the posterior pole. The superotemporal and inferotemporal veins appeared tortuous with few flame-shaped haemorrhages seen. A concentric rim of retinal whitening was seen around the macula, extending temporally and inferiorly, giving rise to a pseudo-cherry-red spot. A few patches of polygonal-shaped retinal whitening were noted. The left fundus showed a hyperaemic optic disc and had similar findings as the right fundus (Fig. 1A, B).

**Fig. 2:**
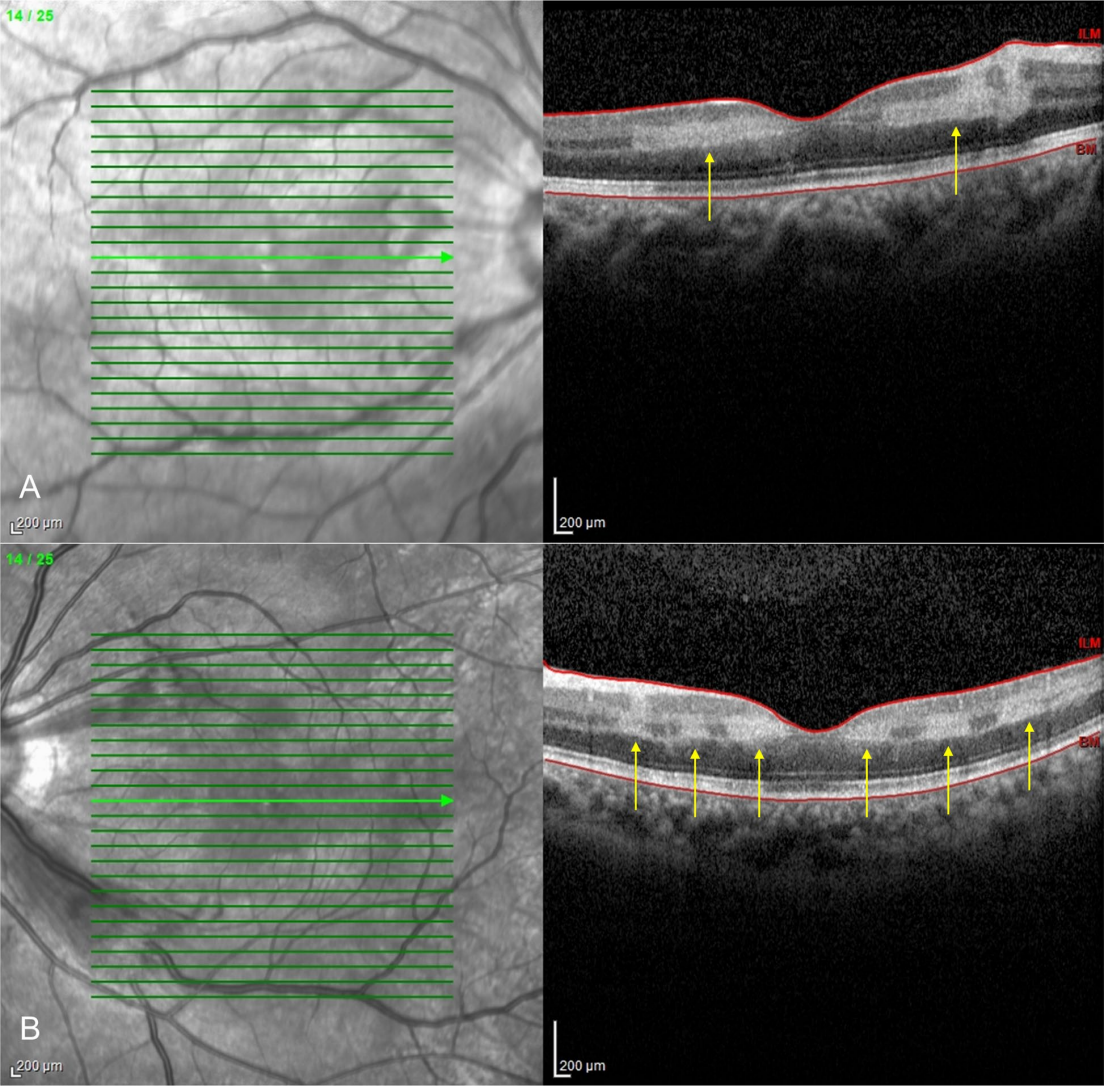
SD-OCT of the right eye (**A**) and the left eye (**B**) showing hyper-reflective bands mainly at the level of the INL (yellow arrows) consistent with the diagnosis of PAMM. The ellipsoid zone and the outer retinal layers appear intact

Spectral domain-optical coherent tomography (SD-OCT) revealed hyper-reflective bands mainly at the level of the inner nuclear layer (INL). At the maculopapular region of the right OCT, these bands extended up to the nerve fibre layer. The outer retinal layers and ellipsoid layers were intact (Fig. 2). Fundus fluorescein angiography (FFA) showed a delay in the arm-to-retina time (31 s), delayed arterial filling (8 s) and prolonged arteriovenous transit time (1:42 min). There was staining of the optic discs and blood vessels at the posterior pole, enlarged foveal avascular zone and capillary non-perfusion perifoveally in both eyes (Fig. 1C–F).

She was diagnosed with bilateral Purtscher-like retinopathy and PAMM secondary to HA breast filler injection. The oral corticosteroids prescribed by the physician were increased to an anti-inflammatory dose and gradually tapered over 8 weeks. Oral acetylsalicyclic acid 150 mg daily was also prescribed.

**Fig. 3:**
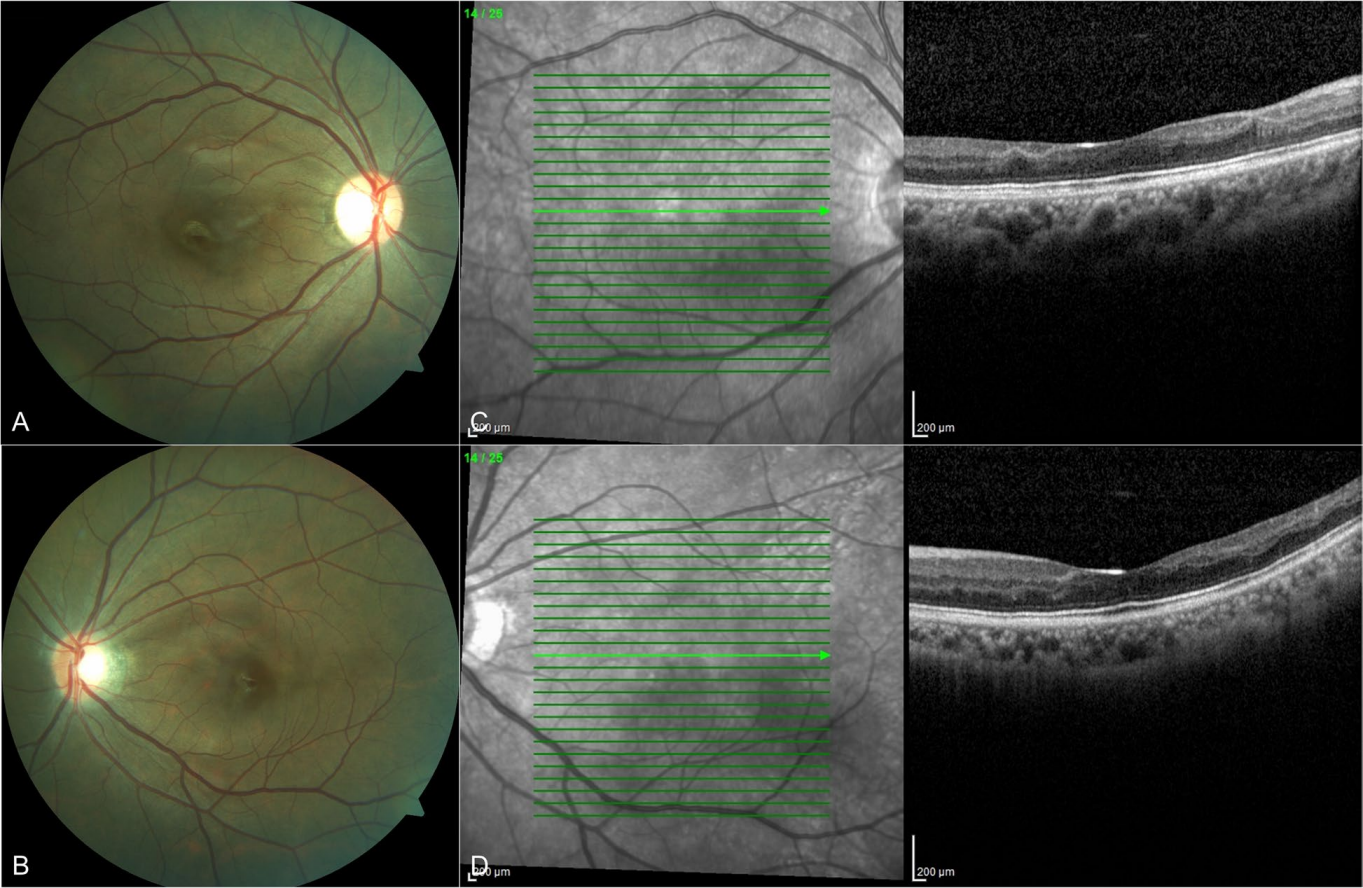
Fundus photograph (**A**, **B**) of both eyes at 10 months follow-up showing resolution of the cotton wool spots and patchy areas of retinal whitening. SD-OCT macula (**C**, **D**) at 10 months follow-up showed thinning of the INL and disorganisation of the retinal inner layers

Three weeks later, the visual acuity improved from counting fingers to 6/18 in both eyes. At 8 weeks, the best corrected visual acuities further improved to 6/12. Serial fundus photography thereafter showed the resolution of the cotton wool spots and patchy areas of retinal whitening. OCT macula showed thinning of the INL and disorganisation of the inner retinal layers (Fig. 3).

## Discussion

**Fig. 4:**
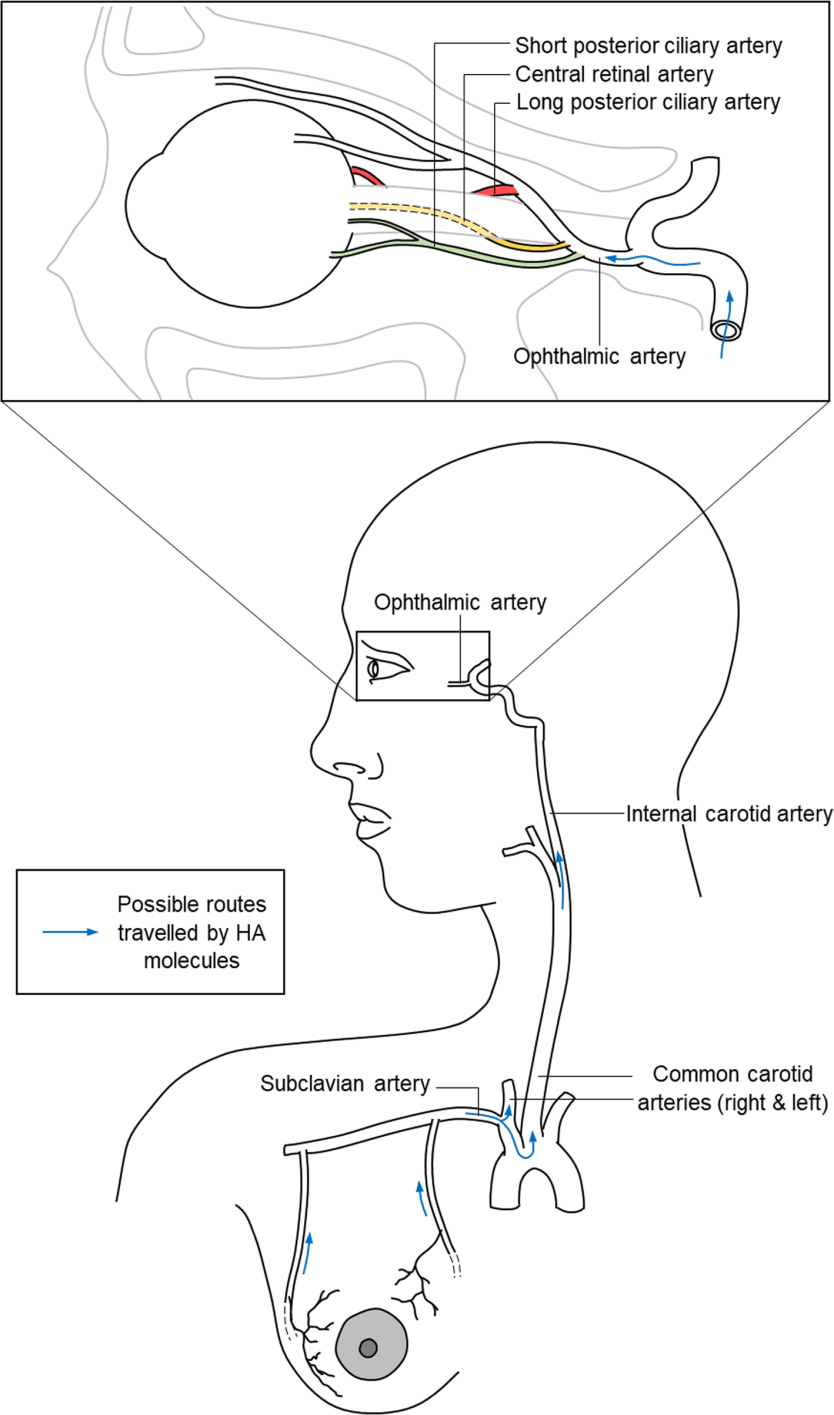
Diagram showing the proposed route of travel of HA molecule from the site of HA injection to ocular blood circulation

We believe this case is the first reported Purtscher-like retinopathy and PAMM following HA fillers involving breast tissue. We postulate that there was an inadvertent inoculation of HA into the artery supplying the breast which then passed on to the subclavian artery, the brachiocephalic trunk and onward to the arch of aorta, ascending to both common carotid arteries and to the internal carotid arteries. HA particles then passed through the ophthalmic arteries, the distal branches of the central retinal artery and finally reached the capillaries and caused occlusion (Fig. 4). The tendency for embolic occlusion is higher in distal arteries than in proximal arteries as the luminal diameter decreases with distality. Besides that, high volumes of filler injection could also predispose to occlusion [5].

In view of the high content of fat in breast tissue, the other possible explanation for this patient's pulmonary and ocular complications could be fat embolism. Fat embolism could result from mechanical obstruction from fragments of fatty tissue causing obstruction in the pulmonary microvascular system. Alternatively, a paradoxical embolism whereby the embolus passes from a patent foramen ovale and enters the arterial circulation, or a microembolism where small emboli pass through the pulmonary artery into the pulmonary and systemic circulation are possible mechanisms. This patient's CECT thorax findings with bilateral ground-glass opacification have also previously been reported in other similar cases of fat embolism syndrome [6].

Although the patient had presented to the ophthalmologist only two weeks later, the clinical features of Purtscher’s retinopathy were apparent in both fundi, mainly the haemorrhages and cotton wool spots in the posterior pole. Small patches of polygonal-shaped retinal whitening also known as Purtscher’s fleckens (Fig. 1A and B) were visible. The SD-OCT confirmed the typical findings of PAMM with increased band-like hyperreflectivity seen in the inner nuclear layer.

PAMM is believed to occur when there is ischemia of the intermediate and deep capillary plexus. The inner nuclear layer and outer plexiform layer may act as a watershed zone being more susceptible to ischaemia. The secondary inflammation provoked by this mechanical occlusion may cause perivascular oedema and further exacerbate hypoperfusion by vaso-compression [7]. Nemiroff et.al found a decrease in vessel density of the deep capillary plexus in PAMM eyes compared with contralateral healthy eyes of the same patients, whereas both PAMM and healthy eyes had equal vessel density of the superficial capillary plexus. In contrast, Kulikov et.al. demonstrated a significant reduction of vessel density in both superficial and deep capillary plexus in PAMM compared to the eyes of healthy volunteers. Furthermore, eyes with PAMM also showed a significant difference in the circularity and the angle of the foveal avascular zone compared with healthy eyes of age-matched controls indicating an alteration of the retinal microvasculature [8, 9].

There were four previous case reports on PAMM following non-facial filler injection. Khatibi reported a case after PMMA injection into buttock muscles, with good visual improvement after a course of systemic steroid therapy [10]; Bruno et.al. reported a similar case following silicone injection in both thighs and buttocks [11]. Table 1 compares our case with other cases of visual loss following non-facial filler injections [12, 13]. To our knowledge, there were no reported cases using HA for breast tissue enhancement presenting with visual loss.

**Table 1:** Comparison of clinical presentation, management, and outcomes with other reported cases of visual loss following non-facial filler injections

*No*	*Authors*	*Age*	*Site of injection*	*Initial VA*		*Time to visual loss*	*Type of filler*	*Ocular diagnosis*	*Systemic associations*	*Management*	*Final VA*	
				*RE*	*LE*						*RE*	*LE*
1	Pee et.al	27	Breast	CF	CF	Same day	Hyaluronic acid	Paracentral acute middle maculopathy, Purtscher-like retinopathy	Diffuse alveolar haemorrhage, toxic encephalopathy	Oral prednisolone	6/12 (20/40)	6/12 (20/40)
2	Huang [12]	20	Gluteal	20/70	20/200	Same day	PMMA	Purtscher-like retinopathy	Diffuse alveolar haemorrhage	Conservative	20/200	20/400
3	Khatibi [10]	32	Buttock	20/40	20/100	3 days	PMMA	Paracentral acute middle maculopathy	Headache, fever, nausea, vomiting, chest tightness	IV antibiotics IV methylprednisolone Oral prednisolone	20/25	20/20
4	Bruno [11]	26	Buttock	20/32	20/63	4–5 days	Silicone	Paracentral acute middle maculopathy, Purtscher-like retinopathy	Silicone embolism syndrome	IV antibiotics IV methylprednisolone Oral prednisolone	Not reported	Not reported
5	Echegaray [13]	31	Penis	20/40	20/20	3 days	Silicone	Paracentral acute middle maculopathy	Silicone embolism syndrome	IV methylprednisolone Oral prednisolone	20/40	20/20

There is no standard evidence-based management for visual loss secondary to filler. However, early and prompt intervention may be potentially sight-saving [14]. Hyaluronidase enzyme is a proposed treatment modality to reverse blindness from HA filler injection [15]. Sharudin et.al. reported a case of full visual recovery following subcutaneous hyaluronidase in a patient with HA-induced visual loss [16]. Carruthers et.al. suggested that early hyaluronidase administration within 60–90 min can disintegrate HA emboli [17]. Hyaluronidase was not used in our patient as she presented two weeks after the onset of symptoms, which is delayed beyond the aforementioned golden period. Furthermore, hyaluronidase was not readily available when the patient presented to us.

Systemic corticosteroids in the form of intravenous or oral steroids have been used in the management to reduce inflammation which may further compromise blood flow. Although our patient presented only after two weeks, she had already received systemic hydrocortisone and oral steroids for the pulmonary involvement. While the visual acuity was still poor at counting fingers, there was gradual visual recovery noted with the continuation of an anti-inflammatory dose of oral corticosteroids. The possibility of this being part of the natural course of PAMM which may be self-limiting cannot be ruled out.

This patient first developed shortness of breath soon after the breast filler procedure and was initially managed by the physicians for life-threatening respiratory distress syndrome. This delayed her referral to the ophthalmologist. Pulmonary complications following HA injections are less common and usually related to non-thrombotic pulmonary embolisms (NTPEs) [18, 19]. Very few cases have reported diffuse alveolar haemorrhage (DAH) the cause of which remains unclear. Some have postulated an immune-mediated response to excessive HA, while others have attributed it to abnormal haemostasis and disruption of the alveolar-capillary integrity [20]. Besides this, she also developed an altered sensorium and was found to have a left corona radiata infarct, raising the possibility of filler-induced cerebral embolism (FICE) which has been reported in the literature [21].

## Conclusions

In summary, we present a case of pulmonary and ocular complications with visual loss due to bilateral Purtscher-like retinopathy and PAMM following HA filler injection to the breast. Systemic and visual recovery was attained following corticosteroid therapy. Practitioners performing filler injections should have good anatomical knowledge and be aware of possible risks to prevent the associated life-threatening and sight-threatening complications.

## Data Availability

All data and materials are available within the paper.

## Abbreviations

CECT: Contrast-enhanced computed tomography
DAH: Diffuse alveolar haemorrhage
EEG: Electroencephalography
FICE: Filler-induced cerebral embolism
FFA: Fundus fluorescein angiography
HA: Hyaluronic acid
INL: Inner nuclear layer
NTPE: Non-thrombotic pulmonary embolisms
OCT: Optical coherent tomography
PAMM: Paracentral acute middle maculopathy
PMMA: Polymethyl methacrylate
